# Transient Global Amnesia (TGA): Younger Age and Absence of Cerebral Microangiopathy Are Potentially Predisposing Factors for TGA Recurrence

**DOI:** 10.3389/fneur.2021.736563

**Published:** 2021-10-27

**Authors:** Andreas Rogalewski, Anne Beyer, Anja Friedrich, Jorge Plümer, Frédéric Zuhorn, Randolf Klingebiel, Friedrich G. Woermann, Christian G. Bien, Isabell Greeve, Wolf-Rüdiger Schäbitz

**Affiliations:** ^1^Department of Neurology, Evangelisches Klinikum Bethel, University Hospital OWL, University Bielefeld, Campus Bielefeld-Bethel, Bielefeld, Germany; ^2^Department of Psychology, Bielefeld University, Bielefeld, Germany; ^3^Department of Neuroradiology, Evangelisches Klinikum Bethel EvKB, University Hospital OWL, University Bielefeld, Campus Bielefeld-Bethel, Bielefeld, Germany; ^4^Department of Epileptology (Krankenhaus Mara), Medical School, Bielefeld University, Bielefeld, Germany

**Keywords:** transient global amnesia, recurrence, cerebral microangiopathy, hypertension, risk factor

## Abstract

**Background:** Transient global amnesia (TGA) is defined by an acute memory disturbance of unclear etiology for a period of less than 24 h. TGA occurs as a single event in most cases. Prevalence rates of recurrent TGA vary widely from 5.4 to 27.1%. This retrospective study aimed to determine predictors for TGA recurrence.

**Methods:** Cardiovascular risk profile and magnetic resonance imaging (MRI) of 340 hospitalized TGA patients between 2011 and 2020 were retrospectively analyzed. The median follow-up period amounted to 4.5 ± 2.7 years. Comparisons were made between TGA patients with and without subsequent recurrence.

**Results:** TGA patients with subsequent recurrence were significantly younger (recurrent vs. single episode, 63.6 ± 8.6 years vs. 67.3 ± 10.5 years, *p* = 0.032) and showed a lower degree of cerebral microangiopathy compared to TGA patients without recurrence. The mean latency to recurrence was 3.0 years ± 2.1 years after the first episode. In a subgroup analysis, patients with at least five years of follow-up (*N* = 160, median follow-up period 7.0 ± 1.4 years) had a recurrence rate of 11.3%. A 24.5% risk of subsequent TGA recurrence in the following five years was determined for TGA patients up to 70 years of age without microangiopathic changes on MRI (Fazekas' score 0).

**Conclusion:** Younger TGA patients without significant microangiopathy do have an increased recurrence risk. In turn, pre-existing cerebrovascular pathology, in the form of chronic hypertension and cerebral microangiopathy, seems to counteract TGA recurrence.

## Introduction

Transient global amnesia (TGA), first described in 1956 (1), is an acute disturbance of memory function for a period of less than 24 h that usually occurs in middle-aged and elderly individuals. The cause of this neuropsychological syndrome with foreground anterograde and retrograde amnesia without focal neurological deficits is thought to reflect a transient deficit in hippocampal function (2) and a reversible disturbance in the functional connectivity of the broader episodic memory network including the medial temporal sub-network, as well as the orbitofrontal-cingulate, medial occipital, inferior temporal and deep-structure sub-networks (3, 4).

Typically, TGA occurs as a single event, but recurrences are occasionally described with up to 11 TGA episodes anecdotally reported in one patient (5). A recent systematic review, evaluating nine cohort studies with a total of 1,989 patients, reported an overall recurrence rate of 13.5% (4), although single studies showed a wide variation (from 5.4 to 27.1%) (6, 7). Interestingly, patients with TGA recurrence were found to be younger (8). There is also evidence for an association between TGA recurrence and a family or personal history of migraine, as well as a personal history of depression (4). Weaker evidence exists for the association with a positive family history of dementia, a personal history of head injury and hippocampal lesions in diffusion-weighted MRI (4).

An association of TGA occurrence with cardiovascular risk factors yielded a lower prevalence of hypertension, diabetes mellitus, dyslipidaemia, and smoking compared to patients with transient ischaemic attack (TIA) (9). Compared to healthy controls, hypertension was associated with TGA only for the more severe stages of the disease, whereas diabetes mellitus (stronger evidence) and smoking (limited evidence) seem to have a protective effect (9). A pathophysiological hypothesis was discussed in which the functional interactions of angiotensin II type-1 and N-methyl-D-aspartate receptors might be of central importance (9).

In a recent study, an association was reported between acute hypertension in patients not adapted to chronic hypertension and the occurrence of TGA (10). TGA patients showed a lower extent of cerebral microangiopathy and less frequent septal hypertrophy in transthoracic echocardiography compared with stroke patients, thus presenting less sequelae of hypertensive heart and brain damage. These observations were supported by a recent review with evidence of similar vascular and mortality risk in TGA patients compared to healthy controls, whereas TIA patients have an increased risk (11).

In contrast, absolute blood pressure values on admission were higher in TGA patients compared with acute stroke patients (10). Based on these findings, it has been hypothesized that failure of cerebrovascular autoregulation (autoregulatory breakdown) and subsequent hypertensive encephalopathy may play a role in the development of TGA, which may occur at lower blood pressure levels in patients unaccustomed to chronic hypertension (10).

The purpose of the present study was to determine predictors for TGA recurrence. Furthermore, we aimed at analyzing the recurrence rate in a large collective with a long follow-up period. In addition, we explicitly addressed the question in our study whether the presence of cerebral microangiopathy and thus hypertrophy of the walls of arterioles is associated with a lower risk of recurrence, since higher blood pressure values are required here to trigger TGA.

## Methods

### Patients

We conducted a retrospective analysis of patient records. A total of 340 patients from one major German hospital with TGA diagnosed by a neurologist according to the criteria defined by Hodges and Warlow (12) were identified between January 1, 2011 and December 31, 2020. Patients who did not meet the diagnostic criteria were not included in the study. This study is an extension of the cohort from a previously reported study (10). Patients treated in 2011, 2012 and 2020 were added so that a full decade could be analyzed.

All patients were included with a discharge diagnosis of TGA during the relevant period. For the identification of possible recurrences, all subsequent inpatient and outpatient contacts were analyzed for these patients, even if the patients presented to the emergency department or outpatient department for other complaints. Evaluation of recurrences was also performed by a neurologist using the diagnostic criteria.

### Procedure

Patient characteristics were evaluated including demographics, cardiovascular risk factors and imaging findings [MRI or computed tomography (CT) scan, ultrasound, echocardiography].

Blood pressure values on admission were recorded and evaluated as single blood pressure values. Chronic hypertension was assumed if it was already indicated by patient history on admission or if antihypertensive medication was required because of hypertensive blood pressure levels during hospital stay and at discharge. Hypertensive blood pressure peaks on admission that normalized during the course and did not require sustained antihypertensive medication were not considered as chronic hypertension.

MRI scans were evaluated for signs of stroke and hippocampal diffusion-weighted imaging (DWI) lesions. The extent of cerebral microangiopathy was assessed using Fazekas' score (0–3) (13) reviewed by a neuroradiologist. The presence of cerebrovascular stenosis was assessed using ultrasound. Laboratory parameters [cholesterol level, glucose level, HbA1c, C-reactive protein (CRP)] were obtained from emergency room records. Echocardiography was evaluated for left ventricular ejection fraction and septal hypertrophy. Septal hypertrophy as a possible indicator for chronic hypertension has been defined as the presence of increased septal thickness (women > 9 mm, men > 10 mm) (14). In addition, CHA2DS2-VASc scores were determined for all patients at the time of discharge. This means that antihypertensive medication at discharge was considered as hypertension in the calculation of the CHA2DS2-VASc score.

Subsequent stays in our hospital, occurring as a result of TGA recurrence, were assessed. Our hospital operates the only department of neurology in town serving a catchment area of about 320,000 people. We therefore assumed that patients with TGA symptoms within this area most probably present to the emergency room of our institution.

### Recurrence Rate

To determine an appropriate recurrence rate, only patients from 2011 to 2015 were considered for this analysis, allowing for a minimum follow-up period of five years.

### Data Analysis

Data analysis was carried out using the Statistical Package for the Social Sciences (SPSS) version 25 (IBM®, International Business Machines Corporation, 2018). Descriptive statistics were displayed as mean ± standard deviation for continuous data and frequencies with percentages for categorical variables. Normal distribution was assessed *via* Shapiro–Wilk test with *p* < 0.05 indicating non-normal distribution, and homoscedasticity was assessed visually *via* q-q-plots.

The profile of cardiovascular risk factors was compared between TGA patients with and without recurrence by using parametric *t*-tests or non-parametric Mann–Whitney *U*-tests, depending on normal distribution. Patient groups (with versus without TGA recurrence) were compared using chi-square tests for categorical variables. A significance level of less than 0.05 in the two-sided test was assumed to be significant. In order to correct for alpha error accumulation in multiple testing, *p*-values were adjusted using the Bonferroni method (p_adj_ = p_obs_^*^k; where p_adj_: adjusted *p*-value, p_obs_: observed *p*-value and *k* = number of comparisons) (15).

## Results

### Demographic Characteristics

**Figure 1:**
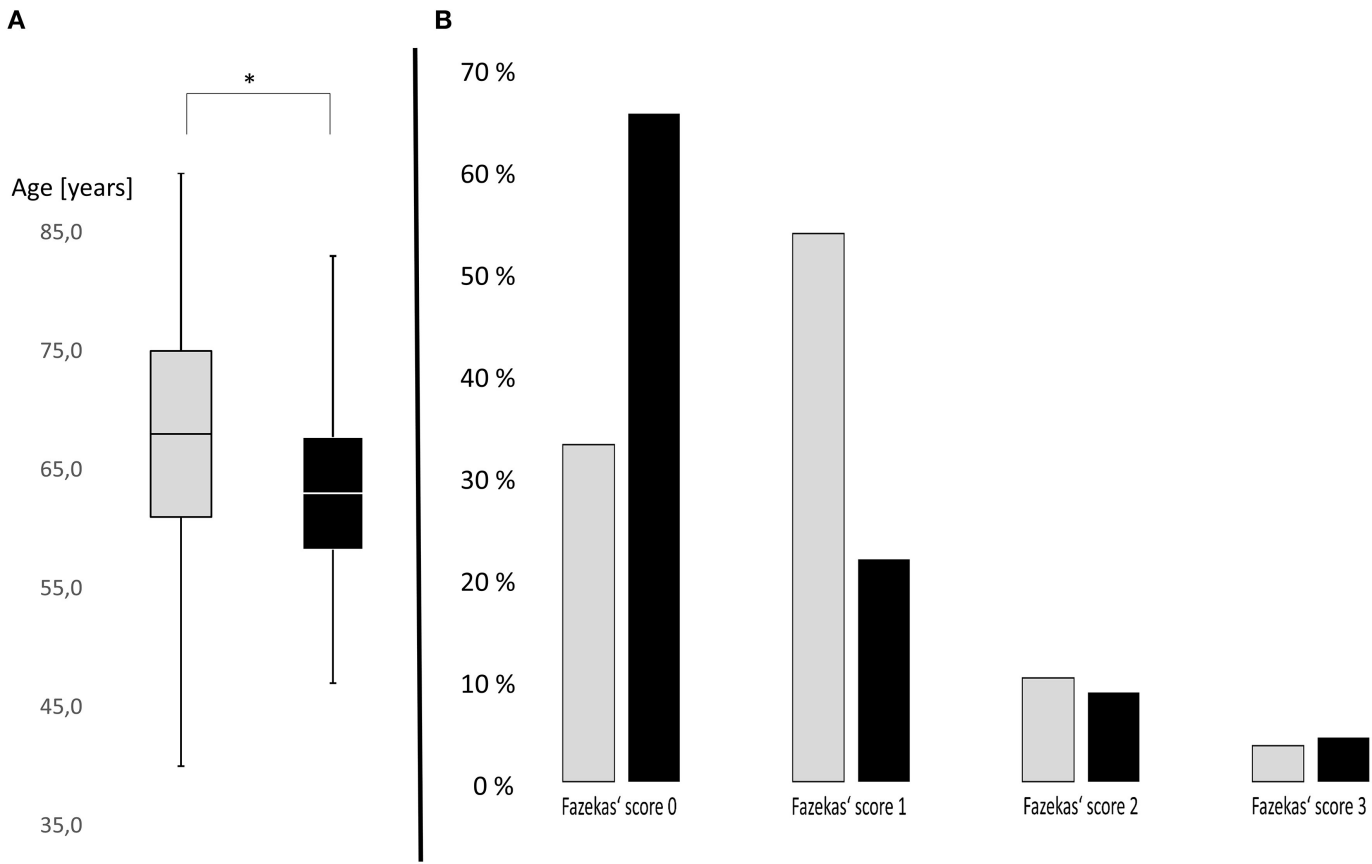
**(A)** Box plots of age distribution in patients with subsequent recurrence (black) and without subsequent recurrence (gray). Transient global amnesia (TGA) patients with subsequent recurrence were significantly younger than patients without recurrence (*p* = 0.032). *Y*-axis: age of patients in years. *p < 0.05. **(B)** Severity distribution of cerebral microangiopathy using Fazekas' score in TGA patients with subsequent recurrence (black) and without subsequent recurrence (gray). TGA patients with subsequent recurrence are more likely to have no microangiopathy, while microangiopathy is more common in TGA patients without subsequent recurrence. *Y*-axis: Distribution in percent.

In total, 340 TGA patients were included in this study. Twenty-four of them had suffered from a documented TGA recurrence and were hospitalized in our clinic. Mean age was 67.3 ± 10.5 years in TGA patients without recurrence and 63.6 ± 8.6 years in TGA patients with recurrence at onset of first episode (see Figure 1A). TGA patients with recurrence were therefore significantly younger than patients without recurrence (Mann–Whitney *U* = 2,797.500, *z* = 2.144, *p* = 0.032). No significant difference for gender was observed. Further demographic characteristics are presented in Table 1.

**Table 1:** Comparison of TGA patients with vs. without subsequent recurrence (*N* = 340).

	**TGA patients with subsequent recurrence (*N* = 24)**	**TGA patients without subsequent recurrence (*N* = 316)**	
**Age**	63.6 ± 8.6	67.3 ± 10.5	***U*** **=** **2,797.500**, ***Z*** **=** **−2.144**, ***p*** **=** **0.032**^c^
Male	9/24 (37.5%)	125/316 (39.7%)	χ^2^ = 0.040, *p* = 0.842^a^
Hypertension	18/24 (75.0%)	226/312 (72.4%)	χ^2^ = 0.074, *p* = 0.786^a^
Systolic blood pressure on admission	169.1 ± 17.6 mm Hg	170.8 ± 23.2 mm Hg	*U* = 1,987.000, *Z* = −0.300, *p* = 0.764^c^
Diastolic blood pressure on admission	92.8 ± 13.5 mm Hg	92.7 ± 13.5 mm Hg	*T* = −0.032, *p* = 0.975^b^
Diabetes mellitus	3/24 (12.5%)	15/314 (4.8%)	χ^2^ = 2.638, *p* = 0.104^a^
Serum glucose level on admission	123.8 ± 25.4 mg/dl	116.7 ± 20.4 mg/dl	*U* = 3,188.000, *Z* = 1.266, *p* = 0.206^c^
HbA1c	5.6 ± 0.6 %	5.5 ± 0.6 %	*U* = 1,954.500, *Z* = −0.179, *p* = 0.858^c^
Hypercholesterolemia (>200 mg/dl at admission)	14/21 (66.7%)	168/267 (62.9%)	χ^2^ = 0.117, *p* = 0.732^a^
Serum cholesterol level on admission	229.1 ± 37.5 mg/dl	216.9 ± 42.9 mg/dl	*T* = -1.176, *p* = 0.241^b^
CRP level on admission	2.3 ± 5.5 mg/l	2.4 ± 5.5 mg/l	*U* = 3,219.000, *Z* = 0.711, *p* = 0.477^c^
LVEF <50%	1/10 (10%)	2/130 (1.5%)	χ^2^ = 3.171, *p* = 0.075^a^
Septal hypertrophy (male >10 mm, female >9 mm)	6/10 (60%)	85/122 (69.7%)	χ^2^ = 0.404, *p* = 0.525^a^
Cerebral stenosis	0/16 (0%)	22/291 (7.6%)	χ^2^ = 1.870, *p* = 0.171 ^a^
Atrial fibrillation	1/24 (4.2%)	23/314 (7.3%)	χ^2^ = 0.337, *p* = 0.561 ^a^
CHA_2_DS_2_-VASc score	2.6 ± 1.4	2.8 ± 1.6	*U* = 3,593.000, *Z* = −0.437, *p* = 0.662^c^
Presence of DWI lesion	12/24 (50.0%)	137/278 (49.3%)	χ^2^ = 0.005, *p* = 0.946 ^a^
Unilateral vs. bilateral lesion in case of presence of DWI lesion	Bilateral 3/12 (25.0%)	Bilateral 31/137 (22.6%)	χ^2^ = 0.035, *p* = 0.851^a^
**Cerebral microangiopathy**	9/24 (37.5%)	183/278 (65.8%)	***χ***^**2**^ **=** **7.656**, ***p*** **=** **0.006**^a^
Antiplatelet therapy at discharge	15/24 (62.5%)	188/316 (59.5%)	χ^2^ = 0.084, *p* = 0.772 ^a^
Oral anticoagulation (OAC) at discharge	2/24 (8.3%)	23/316 (7.3%)	χ^2^ = 0.036, *p* = 0.849 ^a^
Statin therapy at discharge	15/24 (62.5%)	174/316 (55.1%)	χ^2^ = 0.500, *p* = 0.480 ^a^
Antihypertensive drugs at discharge	17/24 (70.8%)	226/316 (71.5%)	χ^2^ = 0.005, *p* = 0.943 ^a^
Former stroke	2/24 (8.3%)	43/316 (13.6%)	χ^2^ = 0.540, *p* = 0.462^a^

a *Chi-square test*,

b *parametric t-test, and*

c *Mann–Whitney U-test used as appropriate. Parameters with significant difference are highlighted in bold*.

### Latency to Recurrence and Recurrence Rate

The mean follow-up period of all TGA patients (*N* = 340) was 4.5 ± 2.7 years [Minimum: 15 days, Maximum: 9.98 years, Median: 4.57 years]. The mean latency to recurrence was 3.0 ± 2.1 years [Minimum: 32 days, Maximum: 7.7 years, Median: 2.60 years] after the first episode. The number of sustained TGA episodes varied from one to four episodes per patient. No significant difference for mean latency to recurrence for gender was observed (male 2.3 ± 2.0 years; female 3.4 ± 2.1 years; Mann–Whitney *U* = 89.000, *z* = 16.771, *p* = 0.215). The annual incidence rate for recurrence was 1.6% per year in the overall collective.

**Figure 2:**
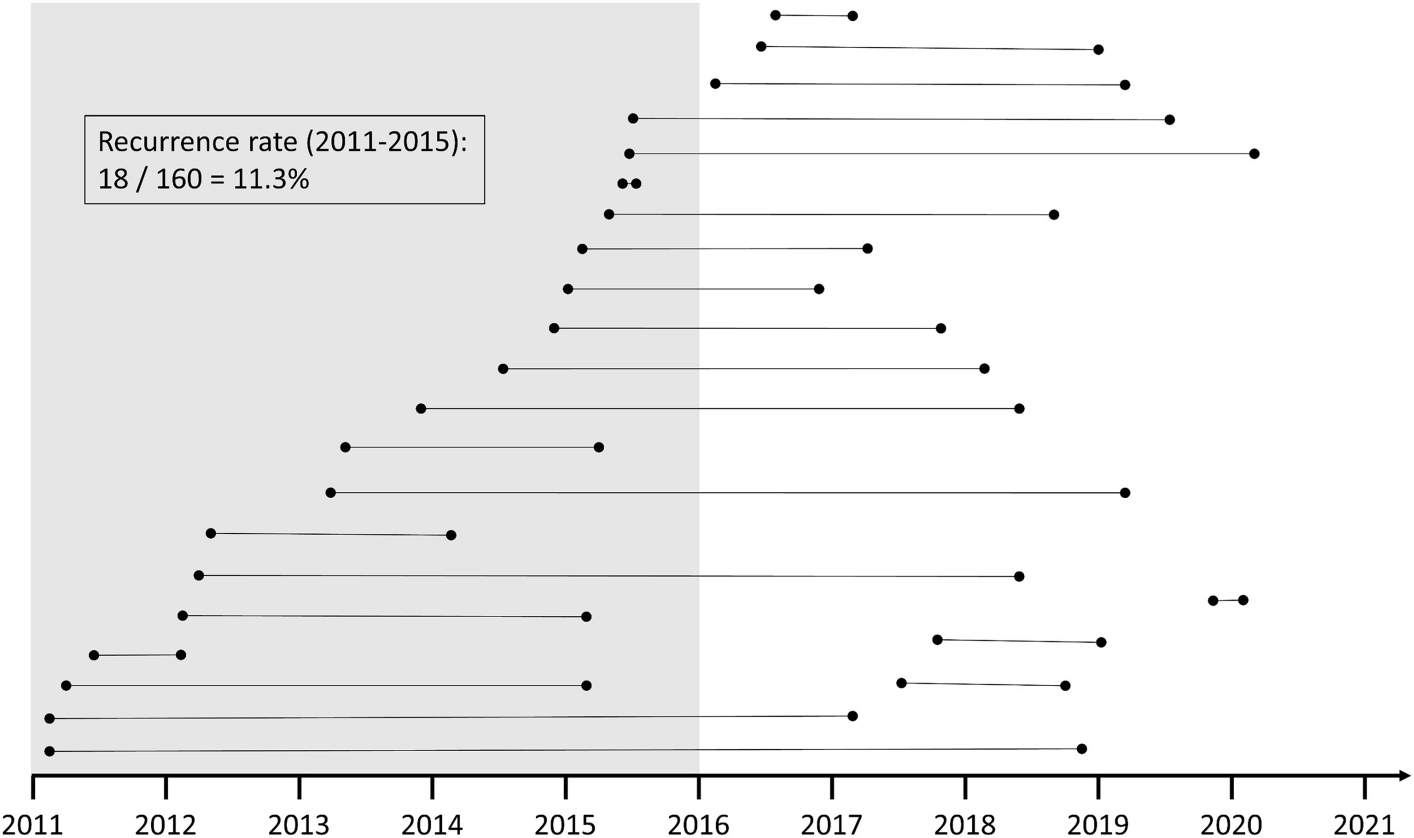
Illustration of the temporal sequence of TGA patients with subsequent recurrence. To assess the recurrence rate, all patients from 2011 to 2015 were chosen as the comparison group to ensure a sufficient duration of the follow-up period.

To determine an appropriate recurrence rate, only patients from 2011 to 2015 were considered for a subgroup analysis. These had at least a five-year follow-up period. The mean follow-up period of this subgroup (*N* = 160) was 7.0 ± 1.4 years [Minimum: 5.1 years, Maximum: 9.98 years, Median: 6.68 years]. During this period, 18 of 160 patients suffered a TGA recurrence corresponding to a recurrence rate of 11.3% (see Figure 2). The annual incidence rate of recurrence in the subgroup with at least a five-year follow-up period was also 1.6% per year.

### Vascular Risk Factors

There were no differences in vascular risk factors on admission (systolic and diastolic blood pressure, cholesterol, HbA1c, CRP, CHA_2_DS_2_-VASc score) between patient groups (isolated vs. recurrent TGA), as assessed by parametric *t*-test (in case of normal data distribution: diastolic blood pressure, cholesterol) or Mann–Whitney *U*-testing (in case of non-normal data distribution: other parameters) (see Table 1). Furthermore, no differences in cardiovascular comorbidity were detected with regard to chronic hypertension, septal hypertrophy in transthoracic echocardiography and hypercholesterolemia. Diabetes mellitus, atrial fibrillation, presence of cerebral stenosis, former stroke and left ventricular ejection fraction (LVEF) in transthoracic echocardiography each had small sample sizes. However, there was no significant difference here either (see Table 1).

### MR Imaging Abnormalities

About 278 of 316 TGA patients without recurrence and all 24 TGA patients with recurrence underwent MRI and were compared. There was no difference between both groups in the presence and number (unilateral versus bilateral) of hippocampal DWI lesions (see Table 1).

The extent of the cerebral microangiopathy was evaluated using the Fazekas' score in available MR images. Fazekas' scoring of available MR images revealed no cerebral microangiopathy (Fazekas' score 0) in 65.2% of TGA patients with recurrence (32.9% of TGA patients without recurrence), mild microangiopathy (score 1) in 21.7% (53.5%), moderate microangiopathy (score 2) in 8.7% (10.1%) and severe microangiopathy (score 3) in 4.3% (3.5%). Distribution of microangiopathic lesions using Fazekas' score in both groups (with versus without recurrence of TGA) is displayed in Figure 1B. Using Mann–Whitney *U*-test, the degree of microangiopathy (ranking from 0 = none to 3 = severe) was significantly lower in TGA patients with recurrence compared to TGA patients without recurrence (*U* = 2,326.000, *z* = 2.394, *p* = 0.017). The effect size according to Cohen was *r* = 0.25 corresponding to a small effect.

Based on an existing correlation between age and extent of cerebral microangiopathy in all patients (Pearson r 0.334, *p* < 0.001), a subgroup analysis of patients ≤ 70 years was performed (Mann–Whitney *U*-test of age between these groups: *U* = 1,771.500, *z* = 0.777, *p* = 0.437). Between groups with recurrent versus isolated TGA in patients aged ≤ 70 years, Fazekas' scoring of available MR images revealed no cerebral microangiopathy (Fazekas' score 0) in 70.0% of TGA patients with recurrence (41.7% of TGA patients without recurrence), mild microangiopathy (score 1) in 20.0% (43.9%), moderate microangiopathy (score 2) in 5.0% (7.1%) and severe microangiopathy (score 3) in 5.0% (2.0%). Using Mann–Whitney *U*-test, the degree of microangiopathy (ranking from 0 = none to 3 = severe) was significantly lower in TGA patients aged ≤ 70 years with recurrence compared to TGA patients without recurrence (*U* = 1,346.000, *z* = 2.045, *p* = 0.041). The effect size according to Cohen was *r* = 0.26 corresponding to a small effect. This analysis demonstrates a difference regardless of age.

In this subgroup of patients aged ≤ 70 years without microangiopathic changes on MRI (Fazekas' score 0) and at least 5 years of follow-up (*N* = 49), 12 patients suffered a recurrence within 5 years (24.5%).

## Discussion

As reported here, patients with TGA recurrence were significantly younger when compared to patients with isolated TGA episodes. The recurrence rate was 11.3% in patients with a follow-up of at least five years; the mean latency to recurrence was 3.0 ± 2.1 years after the first episode. In TGA patients with subsequent recurrence, the extent of cerebral microangiopathy was lower compared to TGA patients without recurrence. A 24.5% risk of subsequent TGA recurrence in the next five years was determined for TGA patients up to 70 years of age without microangiopathic changes on MRI (Fazekas' score 0).

The recurrence rate of our study is very consistent with the rate of 13.5% ascertained from a recent review from nine different cohort studies involving 1,989 patients (4). An overview of studies on recurrence rates in TGA is displayed in Table 2 (6–8, 16–29). The recurrence rates varied between these studies, which might be explained by the diagnostic accuracy of the patients investigated. For example, a disclosure of a TIA as a potential alternative diagnosis was not guaranteed in all cases (17). Furthermore, the duration of the follow-up period ranged widely from 16.5 months (7) to 15.0 years (8).

**Table 2:** Studies and reports on TGA patients with TGA recurrences.

**Study**	**Number of TGA patients**	**Number of TGA recurrences**	**Recurrence rate (mean; time interval)**	**Follow-up-duration (mean; time interval)**	**Results in terms of recurrence risk**	**Limitations**
Hinge et al. (16) Multicenter study	74	16	21.6%	66.6 months	–	
Miller et al. (17) Prospective follow-up	277	66	23.8%	80 months	–	Recurrent episodes included recurrent amnesia, stroke, TIA, migraine, seizure, cardiovascular disease
Fredericks et al. (18)	57	15	26.3%	Not given	–	
Melo et al. (19) Prospective case-control study	51	3	5.9%	17.4 months 01/1985–03/1990	–	Low number of recurrences no comparison of recurrent TGA and single TGA
Gandolfo et al. (20) Prospective follow-up Study	102	19	18.6%	82.2 months	–	No comparison of recurrent TGA and single TGA
Zorzon et al. (21) Case-control study	64	6	9.4%	45.6 ± 37.7 months 1983–1993	–	No comparison of recurrent TGA and single TGA
Klötzsch et al. (22) Case-control study	53	12	23%	Not given 1988–1995	–	no comparison of recurrent TGA and single TGA
Pai and Yang (23) Retrospective study	25	3	12%	45.6 months 07/1988–12/1997	–	No comparison of recurrent TGA and single TGA
Pantoni et al. (24) Case control study	51	4	7.8%	6.8 ± 1.1 years 01/1992–07/1993	–	No comparison of recurrent TGA and single TGA
Agosti et al. (25) Retrospective study	85	12	14.1%	Not given 01/2002–12/2004	Higher frequency of carotid atheromasia and ischemic heart disease. Correlation of Recurrent TGA with higher risk factor sum (number of trigger risk factors)	
Arena et al. (6) Case-control study	221	12	5.4% Mean interval: 4.2 years [2.8–8.4]	12.3 ± 7 years 01/1985–12/2010	–	No comparison of recurrent TGA and single TGA
Larner (26) Author's personally documented consecutive series of TGA	34	9	26%	2002–2016	–	No comparison of recurrent TGA and single TGA
Alessandro et al. (27) Retrospective single center cohort study	203	16	7.9%	22 months 01/2011–03/2017	More frequent history of migraine (37.5% vs. 14%, p=0.03)	
Morris et al. (8) Retrospective cohort study	1,044	143	13.7%	9.4 ± 6.4 years in TGA single episode; 15.0 ± 8.8 years with recurrent episodes 08/1992–02/2018	Recurrent TGA associated with earlier age and higher prevalence of personal and family history of migraine	
Tynas et al. (28) Prospective study	93	15	16.0%	– 2004–2016	Prediction of depression, previous head injury and family history of dementia	
Romoli et al. (29) Comparison of two Independent cohort studies	639	39	6.1%	6.1 years	No differences in risks of recurrent TGA between TGA <1h vs. TGA≥1h	
Oliveira et al. (7) Retrospective study	70	19	27.1%	16.5 mths	Female sex, depression, shorter episode duration, hippocampal hyperintensity on MRI	
Current study 2021 Retrospective study	340	24	7.1% 11.3%	4.5 ± 2.7 years 01/2011–12/2020 7.0 ± 1.4 years 01/2011–12/2015 Follow–up until 12/2020	Younger age, lower extent of cerebral microangiopathy regardless of age	

Our finding that patients with TGA recurrence are younger is supported by previous studies (4). Compared to the previously reported studies, a strength of our study is the large collective and the long follow-up period. Only two reported collectives were larger (8, 30) and had a longer period of follow-up (6, 8).

### Recurrence Risk and MRI Findings

Oliveira et al. reported more punctate hippocampal hyperintensities in patients with recurrent TGA (*n* = 5) versus single TGA (*N* = 0) in a total of 34 patients with available MRI data (*p* = 0.001) (7). In our study, there was no difference between patients with versus without subsequent recurrence in the presence and number (unilateral vs. bilateral) of hippocampal DWI lesions. This result is consistent with other previous studies (8, 27, 28).

A recent study by our group showed an association between acute hypertension and TGA occurrence (10). Herein, TGA patients were less likely to suffer from chronic hypertension compared to acute stroke patients. This was reflected by lower levels of consecutive hypertensive disorders, such as the extent of cerebral microangiopathy and septal hypertrophy in transthoracic echocardiography. These observations are substantiated by evidence of a lower vascular mortality risk in TGA patients compared with TIA patients (11). It was hypothesized that acute hypertensive peaks may trigger TGA episodes, especially in patients who are not adapted to chronic hypertension (10). These observations are well in line with our current results. The risk of recurrence is increased in patients who (1) are younger and (2) have less sequelae of chronic hypertension such as cerebral microangiopathy secondary to wall hypertrophy in arterioles. These patients might be more prone to TGA recurrence as they are less well adapted to episodes of acutely increased blood pressure. The predictor “lack of adaptation to hypertensive blood pressure episodes” persists longer in younger patients and is supported by the absence of microangiopathic lesions, leaving these patients at persistently increased risk for recurrent TGA. It is unclear, however, whether the elevated blood pressure values are a cause or consequence of a TGA episode. One could argue that the elevated acute blood pressure values are a consequence of emotional stress. Alternatively, TGA patients have significantly higher acute blood pressure values on admission compared with acute stroke patients (10), whereas stroke patients may have greater stress.

In clinical practice, patients frequently ask if they will have to face TGA episodes ever again. Our data may assist in patient guidance by informing about the presumptive recurrence risk. Patients with an age up to 70 years and absence of cerebral microangiopathy are subjected to an approximately 25% risk of recurrence within a median latency of four years. Therapeutically, the avoidance of blood pressure peaks would be advisable but cannot be proven by our data. Interestingly, a case report of successful prophylaxis of recurrent coital TGA with metoprolol supports this theory (31). Further prospective studies are required in order to address this issue.

### Study Limitations

Because of the retrospective design, it is possible that not all patients with TGA recurrence were included in our study, since no telephone survey was conducted using recurrence questionnaires. However, our hospital is a tertiary hospital operating the only neurology department including a large Stroke Unit for a catchment area of more than 320,000 inhabitants. TGA patients are typically admitted to our hospital due to presumptive stroke. Therefore, the vast majority of TGA patients in our area should have been treated in our department and subsequently included in the study.

Due to the retrospective study design, the exact time of TGA symptom onset and the resulting time interval between symptom onset and the MRI examination could not be clearly ascertained in all cases. Unfortunately, many of reported TGA predictors, such as personal and family history of migraine, personal history of depression, positive family history of dementia and a personal history of head injury, as well as several conditions under which TGA occurred, such as Valsalva maneuver, medication and sexual intercourse, could not be examined in our retrospective study design in particular because these conditions were not systematically recorded in patient records. This is a weakness of the study due to the retrospective study design. In a prospective study approach, these would be relevant parameters to collect. Due to the lack of a systematic survey of the duration of amnesia as well as limited MRI rates, a rare disease such as transient amnestic epilepsy could not be disclosed in all cases. We believe that this very rare differential diagnosis could be adequately delineated by inpatient workup in a neurology department. Patients with transient epileptic amnesia did not receive a diagnosis of TGA. Because of the lack of systematic follow-up, we have to consider that the older participants had a higher probability of dying within the next 5 years compared to younger participants and therefore did not experience another TGA episode (resulting in an association of younger age and TGA recurrence). It is also possible that some patients with recurrence did not seek neurologic help if it resembled a previous TGA. Here, the behavior in seeking help could possibly differ between younger and older patients. Another limitation of our study is the overall small sample size of TGA recurrence. This leads to a limitation of possible calculations, e.g., association of diabetes and risk of recurrence. The small sample size questions the generalizability of the data.

## Conclusion

Our data support the observation of significant differences in TGA patients with and without recurrence, depending on certain predictors. TGA patients with subsequent recurrence were significantly younger and showed a lower extent of cerebral microangiopathy compared to TGA patients without recurrence. The association between TGA recurrence and cerebral microangiopathy is a novel finding that has not been described previously. It is known that TGA patients are less likely to have chronic hypertension compared to acute stroke patients, which is reflected in lower levels of consecutive hypertensive disorders such as the extent of cerebral microangiopathy. It is reasonable to assume that patients with chronic hypertension are adapting to hypertensive blood pressure figures while carrying a higher risk of secondary diseases such as heart attack and stroke in the long run. Patients without chronic hypertension seem to be more susceptible to hypertensive peaks and are more likely to suffer TGA. Previously normotensive individuals can develop signs of encephalopathy as a result of failure of the upper limit of cerebral vascular autoregulation (autoregulation breakthrough) at blood pressures as low as 160/100 mm Hg, whereas individuals with chronic hypertension may not do so until the blood pressure rises to 220/110 mm Hg or greater (32). An acute blood pressure dysregulation might cause metabolic stress in the hippocampal CA1 sector, which is known for its vulnerability to metabolic and oxidative stress [such as caused by hypoxaemia, β-amyloid-induced neurotoxicity and ischemia-mediated glutamate overload and calcium influx (33, 34)]. Cerebral microangiopathy as a marker of hypertrophy of the arterioles thus represents a possible protective factor that may not prevent the occurrence of TGA but may allow it to occur predominantly at higher blood pressure levels. This may be an explanation for our finding of increased TGA recurrence risk in younger patients and patients without sequelae of chronic hypertension. This theory may also explain the observation of vascular risk factors, why hypertension was associated with TGA only for the more severe stages of the disease compared with healthy controls, whereas diabetes mellitus (stronger evidence) and smoking (limited evidence) seem to have a protective effect (9). Our retrospective study design does not allow for proving a causal relationship between TGA recurrence risk and acute hypertensive episodes but highlights adaptation to hypertension as a potential impact factor.

## Data Availability Statement

The raw data supporting the conclusions of this article are available from the corresponding author upon reasonable request.

## Ethics Statement

Our study complied with the guidelines for human studies and was conducted ethically in accordance with the Declaration on Ethics of the World Medical Association of Helsinki. The study was approved by the Local Ethics Committee of Muenster (file reference: 2021-288-f-S). Written informed consent for participation was not required for this study in accordance with the national legislation and the institutional requirements because this study was a retrospective analysis of patient records.

## Author Contributions

AR, AB, and WS designed the study and drafted the manuscript. AR, AB, and JP performed data acquisition. AR and AF performed statistical analysis. RK and FW performed MRI analysis. FZ, IG, RK, and CB revised the manuscript. All authors were involved in data evaluation and discussions.

## Funding

AB received funding by the Society for Epilepsy Research Bethel.

## Conflict of Interest

The authors declare that the research was conducted in the absence of any commercial or financial relationships that could be construed as a potential conflict of interest.

## Publisher's Note

All claims expressed in this article are solely those of the authors and do not necessarily represent those of their affiliated organizations, or those of the publisher, the editors and the reviewers. Any product that may be evaluated in this article, or claim that may be made by its manufacturer, is not guaranteed or endorsed by the publisher.

## Abbreviations

CT: computed tomography
DWI: diffusion-weighted imaging
MRI: magnetic resonance imaging
TGA: transient global amnesia
TIA: transient ischaemic attack.

## References

[1] Bender MB. Syndrome of isolated episode of confusion with amnesia. J Hillside Hosp. (1956) 5:212–5.5596323

[2] Eugenia Marin-Garcia. Neurocognitive Perspective of Transient Global Amnesia, Neurological and Mental Disorders, Kaneez Fatima Shad and Kamil Hakan Dogan, IntechOpen. London, UK: IntechOpen (2019). 10.5772/intechopen.88810

[3] Park KM Lee BI Kim SE. Is transient global amnesia a network disease? Eur Neurol. (2018) 80:345–54. 10.1159/000496511 30928984

[4] Liampas I Raptopoulou M Mpourlios S Siokas V Tsouris Z Aloizou AM. Factors associated with recurrent transient global amnesia: systematic review and pathophysiological insights. Rev Neurosci. (2021) 32:751–65. 10.1515/revneuro-2021-0009 33675214

[5] Saleem S Patel R Gawarikar Y. A rare case of recurrent transient global amnesia. J Neurol Neurosurg Psychiatry. (2018) 89:A38.3–A39. 10.1136/jnnp-2018-ANZAN.96

[6] Arena JE Brown RD Mandrekar J Rabinstein AA. Long-term outcome in patients with transient global amnesia: a population-based study. Mayo Clin Proc. (2017) 92:399–405. 10.1016/j.mayocp.2016.11.015 28185658 PMC5682935

[7] Oliveira R Teodoro T Marques IB. Risk factors predicting recurrence of transient global amnesia. Neurol Sci. (2021) 42:2039–43. 10.1007/s10072-020-04788-6 33033897

[8] Morris KA Rabinstein AA Young NP. Factors associated with risk of recurrent transient global amnesia. JAMA Neurol. (2020) 77:1551–8. 10.1001/jamaneurol.2020.2943 32865551 PMC7489420

[9] Liampas I Raptopoulou M Siokas V Bakirtzis C Tsouris Z Aloizou AM. Conventional cardiovascular risk factors in transient global amnesia: systematic review and proposition of a novel hypothesis. Front Neuroendocrinol. (2021) 61:100909. 10.1016/j.yfrne.2021.100909 33539928

[10] Rogalewski A Beyer A Friedrich A Plümer J Zuhorn F Greeve I. Transient global amnesia (TGA): influence of acute hypertension in patients not adapted to chronic hypertension. Front Neurol. (2021) 12:1003. 10.3389/fneur.2021.666632 34305782 PMC8296302

[11] Liampas I Raptopoulou M Siokas V Tsouris Z Brotis A Aloizou A. The long-term prognosis of transient global amnesia: a systematic review. Rev Neurosci. (2021) 32:531–43. 10.1515/revneuro-2020-0110 33550779

[12] Hodges JR Warlow CP. Syndromes of transient amnesia: towards a classification. A study of 153 cases. J Neurol Neurosurg Psychiatry. (1990) 53:834–43. 10.1136/jnnp.53.10.834 2266362 PMC488242

[13] Fazekas F Chawluk JB Alavi A Hurtig HI Zimmerman RA. MR signal abnormalities at 1.5 T in Alzheimer's dementia and normal aging. Am J Roentgenol. (1987) 149:351–6. 10.2214/ajr.149.2.351 3496763

[14] Lang RM Badano LP Victor MA Afilalo J Armstrong A Ernande L. Recommendations for cardiac chamber quantification by echocardiography in adults: an update from the american society of echocardiography and the European association of cardiovascular imaging. J Am Soc Echocardiogr. (2015) 28:1–39.e14. 10.1016/j.echo.2014.10.003 25559473

[15] Bland JM Altman DG. Multiple significance tests: the Bonferroni method. BMJ. (1995) 310:170. 10.1136/bmj.310.6973.170 7833759 PMC2548561

[16] Hinge HH Jensen TS Kjaer M Marquardsen J Fine Olivarius B. The prognosis of transient global amnesia: results of a multicenter study. Arch Neurol. (1986) 43:673–6. 10.1001/archneur.1986.00520070031013 3729744

[17] Miller JW Petersen RC Metter EJ Millikan CH Yanagihara T. Transient global amnesia: clinical characteristics and prognosis. Neurology. (1987) 37:733–7. 10.1212/WNL.37.5.733 3574671

[18] Fredericks JMH. Transient global amnesia and related disorders. Markowitsch HJ. Transient Global Amnesia and Related Disorders. Ashland, OH, US: Hogrefe & Huber Publishers. (1990). p. 28–47.

[19] Melo TP Ferro JM Ferro H. Transient global amnesia: a case control study. Brain. (1992) 115:261–70. 10.1093/brain/115.1.261 1559158

[20] Gandolfo C Caponnetto C Conti M Dagnino N Del Sette M Primavera A. Prognosis of transient global amnesia: a long-term follow-up study. Eur Neurol. (1992) 32:52–7. 10.1159/000116787 1563456

[21] Zorzon M Antonutti L Mase G Biasutti E Vitrani B Cazzato G. Transient global amnesia and transient ischemic attack: natural history, vascular risk factors, and associated conditions. Stroke. (1995) 26:1536–42. 10.1161/01.STR.26.9.1536 7660394

[22] Klötzsch C Sliwka U Berlit P Noth J. An increased frequency of patent foramen ovale in patients with transient global amnesia. Analysis of 53 consecutive patients. Arch Neurol. (1996) 53:504–8. 10.1001/archneur.1996.00550060046014 8660151

[23] Pai MC Yang SS. Transient global amnesia: a retrospective study of 25 patients. Zhonghua Yi Xue Za Zhi (Taipei). (1999) 62:140–5. 10222601

[24] Pantoni L Bertini E Lamassa M Pracucci G Inzitari D. Clinical features, risk factors, and prognosis in transient global amnesia: a follow-up study. Eur J Neurol. (2005) 12:350–6. 10.1111/j.1468-1331.2004.00982.x 15804264

[25] Agosti C Akkawi NM Borroni B Padovani A. Recurrency in transient global amnesia: a retrospective study. Eur J Neurol. (2006) 13:986–9. 10.1111/j.1468-1331.2006.01408.x 16930365

[26] Larner AJ. Recurrent transient global amnesia: is there a link to familial history? Prog Neurol Psychiatry. (2017) 21:17–9. 10.1002/pnp.481

[27] Alessandro L Calandri IL Suarez MF Heredia ML Chaves H Allegri RF. Transient global amnesia: clinical features and prognostic factors suggesting recurrence. Arq Neuropsiquiatr. (2019) 77:3–9. 10.1590/0004-282x20180157 30758436

[28] Tynas R Panegyres PK. Factors determining recurrence in transient global amnesia. BMC Neurol. (2020) 20:83. 10.1186/s12883-020-01658-8 32143587 PMC7060647

[29] Romoli M Tuna MA Li L Paciaroni M Giannandrea D Tordo Caprioli F. Time trends, frequency, characteristics and prognosis of short-duration transient global amnesia. Eur J Neurol. (2020) 27:887–93. 10.1111/ene.14163 32012408 PMC7115816

[30] Romoli M Tuna MA McGurgan I Li L Giannandrea D Eusebi P. Long-term risk of stroke after transient global amnesia in two prospective cohorts. Stroke. (2019) 50:2555–7. 10.1161/STROKEAHA.119.025720 31284848

[31] Berlit P. Successful prophylaxis of recurrent transient global amnesia with metoprolol. Neurology. (2000) 55:1937–8. 10.1212/WNL.55.12.1937 11134409

[32] Vaughan CJ Delanty N. Hypertensive emergencies. Lancet. (2000) 356:411–7. 10.1016/S0140-6736(00)02539-3 10972386

[33] Kosuge Y Imai T Kawaguchi M Kihara T Ishige K Ito Y. Subregion-specific vulnerability to endoplasmic reticulum stress-induced neurotoxicity in rat hippocampal neurons. Neurochem Int. (2008) 52:1204–11. 10.1016/j.neuint.2007.12.010 18280615

[34] Bartsch T Deuschl G. Transient global amnesia: functional anatomy and clinical implications. Lancet Neurol. (2010) 9:205–14. 10.1016/S1474-4422(09)70344-8 20129169

